# Assessment of Paediatricians' Oral Health Knowledge, Behaviour, and Attitude in the United Arab Emirates

**DOI:** 10.1155/2020/7930564

**Published:** 2020-09-22

**Authors:** Noora Aburahima, Iyad Hussein, Mawlood Kowash, Anas Alsalami, Manal Al Halabi

**Affiliations:** ^1^Dubai Health Authority, Dubai, UAE; ^2^Pediatric Dentistry Department, Hamdan Bin Mohammed College of Dental Medicine, Mohammed Bin Rashid University of Medicine and Health Sciences, P.O. Box 505055, Dubai, UAE

## Abstract

**Aim:**

Paediatricians' oral health knowledge is essential for early detection of disease, appropriate advice, and proper referral. Therefore, this study aimed to evaluate the knowledge, behaviour, and attitude of paediatricians practicing in the United Arab Emirates (UAE) regarding children's oral health. *Study Design*. Cross-sectional survey.

**Methods:**

A questionnaire consisting of 16 questions was completed after piloting by a sample of randomly selected registered UAE paediatricians. Scores of knowledge (out of 9), behaviour (out of 4), and attitude (out of 3) were calculated. Statistical analysis was performed using Shapiro–Wilk, Mann–Whitney *U*, and Kruskal–Wallis tests. Statistical significance was set as *P* < 0.05.

**Results:**

A total of 185 surveys were completed. Nearly half of the paediatricians (95 (51.4%)) identified the appropriate age for child's first dental visit; while 88 (47.6%) believed that the appropriate age to start brushing was after the eruption of the primary molars (2-3 yrs), and 132 (71.4%) believed that the ideal time to give sugary snacks is in between meals. 123 (66.5%) participants said that they would prescribe antibiotics to treat local dental sepsis without fever. Experienced paediatricians and those trained in Western countries had significantly better knowledge about oral health (*P* values 0.040 and 0.031 consecutively). The scores of attitude, behaviour, and knowledge were correlated, and a positive relationship between the scores of knowledge and behaviour was found (*r* = 0.241, *P*=0.001) and between scores of attitude and behaviour (*r* = 0.197, *P*=0.007).

**Conclusions:**

The results demonstrated a general lack of knowledge of oral health aspects by UAE paediatricians. Furthermore, continuous education in the subject is recommended.

## 1. Introduction

The World Health Organization (WHO) defines health as “a state of complete physical, mental, and social wellbeing and not merely the absence of disease or infirmity” [1]. Oral health cannot be detached from general health, and it is considered fundamental to the quality of life [2]. Dental caries is the most prevalent chronic childhood disease, which adversely affects the oral health of infants and children [3]. A decline in caries prevalence in the permanent dentition had been reported, while the prevalence of decay in the primary dentition continues to increase [4]. Carious lesions, if abandoned, can lead to disturbances in growth and development, pain, and serious infections [5].

Early childhood caries (ECC) is a chronic, infectious disease affecting the primary teeth [6]. It is defined as the occurrence of one or more decayed, filled, or missing tooth surfaces in any primary tooth in a child, 6 years of age or younger [7]. Globally, WHO reports caries prevalence in school-age children at 60–90% and as virtually universal among adults in most countries [8].

In the United Arab Emirates (UAE), in 5 years old children, the prevalence was reported to be 82%–94% in the Emirate of Abu Dhabi [9], 83% in the Emirates of Ajman and Dubai [10], and 74.1% in the Emirate of Ras Al-Khaimah [11].

ECC has multiple predisposing factors and early signs that should be recognised in infants in order to provide appropriate preventive measures and to avoid the development of dental caries later in life [9]. These factors include poor dietary feeding habits and inconsistent oral hygiene practices. Moreover, socioeconomic factors such as parental education, family income, and number of siblings are considered leading markers of caries risk and can play a vital role in predicting future dental caries in children [12].

Parents interact frequently with paediatricians during the child's early life. Children visit a paediatrician an average of eight times in their first year of life and 13 times by age of three [13]. Thus, paediatricians are considered a perfect and reliable source to start the caries risk assessment, prevention, and referral of children for dental care when appropriate [14].

For paediatricians to be most successful in promoting oral health and providing prevention guidance, they must acquire current knowledge and understanding of evidence-based oral health preventive measures [15]. In May 2003, the American Academy of Pediatrics (AAP) emphasised that paediatricians and paediatric healthcare professionals should be competent to perform oral health risk assessments on all patients from six months of age [13]. However, several international and regional studies stated that the oral health knowledge capability and practice of many paediatricians is not satisfactory [14–18]. Therefore, the purpose of this study was to assess UAE paediatricians' knowledge, attitude, and behaviour regarding children's oral health. The results might be helpful to determine certain areas where knowledge of paediatricians could be enhanced through additional training and raising awareness.

## 2. Materials and Methods

This study used a cross-sectional design. Data were collected by means of a standardised questionnaire adopted with modifications from Sabbagh et al. and Weatherspoon et al. studies [14, 15]. The target size of the sample of the survey was 176, which represented 10% of the total paediatricians practicing in the UAE according to the information provided by the Federal Competitiveness and Statistics Authority [19]. The authors decided to target 10% of the paediatricians' in the UAE in this study to have an adequate representation of this specific group of practitioners. The survey was first piloted on 10 paediatricians prior to electronic mailing. After receiving feedback, adjustments and revisions were made.

The questionnaire consisted of three parts. The first part included demographic questions about gender, years of experience, professional title, practice setting, Emirate of practice, and country of specialisation. The second part included two sections, the first of which was the dental knowledge section, which included nine questions with either yes/no or multiple choice answers, and the second was the behaviour and practice section that included four questions with yes/no or multiple choice questions. The last part included three questions with yes/no answers about the dental attitude of the participating paediatricians.

The survey was electronically mailed in January 2019 to randomly selected UAE licensed and registered paediatricians (residents, specialists, and consultants), in all health authorities of Dubai Health Authority (DHA), Dubai Healthcare City Authority (DHCA), Health Authority of Abu Dhabi (HAAD), and Ministry of Health (MOH) using Survey Monkey® (http://www.surveymonkey.com). The survey was mailed to every fourth paediatrician on the lists provided. The total number of electronically mailed surveys was 440. The paediatricians in this study were informed that their participation was completely voluntary, and that their participation in the study entails their consent. A second reminder to complete the survey was sent to the selected paediatricians two weeks after the first e-mail.

Statistical analysis included descriptive statistics in addition to cross-tabulation of paediatricians' knowledge, behaviour, and attitude scores of children's oral health. All statistical analyses were conducted using IBM SPSS for windows version 23.0 (IBM Corporation, NY, USA). Statistical analysis was performed using the Shapiro–Wilk test, Mann–Whitney *U* test, and Kruskal–Wallis test. Statistical significance was set as *P* < 0.05.

An approval to conduct the study was obtained from the Research and Ethics Committee at Mohammed Bin Rashid University of Medicine and Health sciences (EC1115-003).

## 3. Results

### 3.1. Demographic Characteristics

Of the four hundred and forty surveys electronically mailed to registered paediatricians, 185 were returned resulting in a response rate of 42%.  Table 1 displays the demographic characteristics of the paediatricians who participated in the study.

### 3.2. Overall Knowledge of Paediatricians regarding Oral Health of Children

Nine questions were asked regarding oral health knowledge. A summary of the questions and results of the answers of the paediatricians to each question is presented in Table 2. Almost half of the paediatricians (95 (51.4%)) correctly chose that the child's first dental visit should be by the age of 6–12 months. Nearly, all paediatricians (173 (93.5%)) recognised that a child with no cavities needs to visit a dentist regularly. Almost half of the paediatricians (88 (47.6%)) assumed that the most appropriate age for children to start brushing their teeth with fluoridated toothpaste is after the eruption of primary molars (2-3 years), while 77 (41.6%) paediatricians were aware of the correct time to start brushing with fluoridated toothpaste as soon as the first tooth erupts (6–12 months). Regarding the best time to give a child a sugary snack, 132 (71.4%) paediatricians wrongly selected in between meals as the best time to give a child a sugary snack.

Regarding early carious lesions without cavitation, 97 (52.4%) paediatricians correctly assumed that these lesions can be remineralised or healed. As for the most appropriate treatment for a 5-year-old child with fever, extraoral swelling, and intraoral swelling, most of the paediatricians (159 (85.9%)) were aware that, in this case, they would prescribe antibiotics and refer to a dentist immediately. Another similar scenario was given about a five-year-old child with a localised dental abscess and no fever. Only 62 (33.5%) correctly chose to prescribe analgesics only and refer the child to a dentist immediately.

The last two questions were about choosing the most appropriate diagnosis for two different scenarios where images were provided. The first scenario was about a 10-day-old infant with an image of natal/neonatal teeth. More than half of the paediatricians (105 (56.8%)) were able to diagnose it appropriately as natal/neonatal teeth. The second question was about a 9-month-old infant with an image of an eruption cyst, 96 (51.9%) paediatricians were able to diagnose it as an eruption cyst/hematoma.

Using the percentages of correct answers to knowledge questions displayed in Table 2, mean scores of knowledge related to children's oral health and prevention were calculated. Scores were based on the number of correct/incorrect answers given by the paediatricians. The mean score of knowledge of all participants was found to be 4.9 out of 9 (SD ± 1.66).

### 3.3. Correlation between Score of Knowledge and Demographics

Table 3 illustrates the correlation between the score of knowledge and the demographic characteristics of the participants. A statistically significant difference was found between the years of experience and the score of knowledge. The mean score of knowledge of paediatricians who had more than or equal to twenty-one years of experience was 6 (±1.38), higher than all those in the rest of the categories (*P*=0.04). Moreover, paediatricians who completed their specialty training in the Western countries including countries in Europe, United Kingdom, North and South America, had a higher score of knowledge, 6 (±1.58), when compared to those who completed their specialty training in the Arab countries that belong to the Arab League and the Indian Subcontinent countries including India, Pakistan, Bangladesh, Nepal, Sri Lanka, Bhutan, and the Maldives (*P*=0.031). No other statistical significance was found when correlating other demographics with the score of knowledge.

### 3.4. Paediatricians' Behaviour

Four questions were asked investigating paediatricians' behaviour toward oral health. A summary of the questions and results of the answers of the paediatricians to each question is presented in Table 4. The overall mean score of behaviour was 2.5 (±0.88) out of 4 based on the correct answers given by the participants for the behaviour questions. For question 4, the participant was given a score of 1 for both answers of “No rewards” and “Others.”

### 3.5. Paediatricians' Attitude

Three questions in the survey investigated paediatricians' attitude toward oral health. A summary of the questions and results of the answers of the paediatricians to each question is presented in Table 5. Based on the predetermined preferred answers given by the paediatricians, the mean score of attitude was found to be 1.96 (±0.85) out of a score of 3.

### 3.6. Correlation between the Scores of Attitude, Behaviour, and Knowledge

When the scores of attitude, behaviour, and knowledge were correlated, there was no significant relationship between the score of knowledge and attitude (*r* = 0.129, *P*=0.079). On the other hand, a positive relationship between the scores of knowledge and behaviour was found (*r* = 0.241, *P*=0.001) and between scores of attitude and behaviour (*r* = 0.197, *P*=0.007).

### 3.7. Previous Oral Health Education

Most of the paediatricians (149 (81.4%)) reported that they did not attend any lectures related to children's oral health. Moreover, almost all paediatricians (170 (91.9%)) reported that they are willing to attend lectures on oral health of children.

## 4. Discussion

This study assessed paediatricians' oral health-related knowledge in the UAE. The results demonstrated a general lack of knowledge of oral health aspects by UAE paediatricians. Oral health is an integral part of general health and wellbeing at every stage of life. Given this relationship, the WHO advocated that oral health should be incorporated into general health promotion and disease prevention practices [20]. Integrating oral health into general health can improve access to oral health care. In order to be most successful in promoting oral health and providing prevention guidance, paediatricians must acquire current knowledge and understanding of evidence-based oral health preventive measures [15].

About half of the surveyed paediatricians (51.4%) were able to identify the ideal timing of the child's first dental visit to be at 6–12 months of age. These results were similar to the results by Oge et al. who reported that 43.8% of their study participants recommended the first dental visit to be at age one [21], while in neighbouring Saudi Arabia, Sabbagh et al. reported a much lower percentage of 25.6% of their study participants who identified that the first dental visit should occur within 6–12 months of age [14]. Although our surveyed paediatricians displayed similar or better knowledge compared to their peers, better knowledge and appreciation of the importance of early dental visits are needed.

Since paediatricians play a pivotal role in providing dietary counselling, they should thoroughly understand the relationship between dental caries and diet to educate their patients regarding cariogenic diets. In our study, 88.5% of the paediatricians reported including information regarding cariogenic food in their dietary counselling to their patients. In comparison, Sabbagh et al. reported that only 30% of their participants provided cariogenic food-related dietary counselling to their patients in neighbouring Saudi Arabia [14]. Interestingly, in the current study, almost half of the paediatricians (48.1%) who rewarded patients at the end of the child's visit rewarded them with sweets. This behaviour would undermine the impact of their dietary instructions, and proper education regarding this issue should be provided to the paediatricians.

The frequent intake of carbohydrate-containing snacks between meals is known to increase the risk of dental caries especially for those individuals with low oral clearance rates [22]. Therefore, highly acidogenic sugar containing snack foods should be consumed at mealtimes to reduce the risk of dental caries and between-meal snacks should be either nonacidogenic or hypoacidogenic [22]. Among the participants of our study, 71.4% believed that sugary snacks should be given in between meals.

The first stage of dental caries to be noticeable is the “white spot” precavitation lesion stage. There is an opportunity for these early carious lesions to heal if the surface layer is unbroken [23]. Paediatricians can detect these early carious lesions during their routine examination of the oral cavity, provide some oral hygiene instructions, and refer to a dentist [14]. Approximately half of the participants of this study agreed with the possibility of early carious lesions to be healed or remineralised like a previous study by Weatherspoon et al., which reported that 41% of the paediatricians agreed that early carious lesions can be remineralised [15]. An issue worth pointing out here is that paediatricians might be able to recognise an early noncavitated lesion, but it might be more challenging for them to detect the “white spots.”

We found it encouraging that many of the surveyed paediatricians demonstrated a good degree of awareness regarding their role in monitoring and promoting oral health care, as 68% of the paediatricians reported that they routinely examine the oral cavity. Although impressive, these results can be influenced by social desirability and should be interpreted with caution. These results are similar to those reported by Sabbagh et al. and Gupta et al. [14, 18]. Gezgin et al. reported that only 44.8% of the study participants examined the oral cavity in the presence of a problem [24]. As mentioned before, social desirability, in addition to cultural and educational factors, might have resulted in these differences.

Antibiotics are considered the most common medication prescribed for children [25]. Recently, the uncontrolled rise in antimicrobial-resistant infections had led to increased morbidity, mortality, and healthcare costs [25]. When the surveyed paediatricians were given a scenario of a 5-year-old child with a localised dental abscess and no fever, 66.5% of them chose to unnecessarily prescribe antibiotics and refer the patient to the dentist. This issue is of great concern and requires proper education. A localised dental infection-related abscess does not require systemic antibiotics [26].

According to the AAP, paediatricians should recommend the use of fluoridated toothpaste to parents as soon as the first tooth erupts [13]. More than half of the surveyed paediatricians (62.8%) recommended the use of fluoridated toothpaste for children. In contrast, Sezer et al. [27] reported that 72.6% of the paediatricians believed that fluoridated toothpaste should not be used in children less than three years of age based on the fact that several studies considered toothpaste swallowing as a risk factor for dental fluorosis [28–30].

When respondents' scores of knowledge, attitude, and behaviour were correlated in this study, analysis demonstrated that behaviour was significantly related to knowledge and attitude.

Even though there was no significant relationship between the scores of knowledge and attitude, yet this relationship can be interpreted as transitive through the behaviour. In other words, paediatricians who had better knowledge regarding oral health demonstrated enhanced behaviour scores, leading eventually to an improved attitude. Although Sabbagh et al. calculated the scores of knowledge, attitude, and behaviour, no measurement of correlation between these scores was performed in their study [14]. Al-Shunairber et al. reported that although most of their participants demonstrated good dental attitude and knowledge, lack of associated practice was detected [17].

In the current study, we found that those who had more than or equal to twenty-one years of experience had significantly better knowledge compared to those with less than 20 years of experience. This supports the theory that experience enhances the knowledge [31]. Sabbagh et al. found out that paediatricians' experience from long periods of practice improved their dental behaviour [14]. In contrast to that, Oge et al. reported that years of experience was associated with a significant decrease in oral health knowledge. Participants of that study with nine or less years of experience had higher level of oral health knowledge [21].

Paediatricians trained in the Western countries in this study demonstrated better knowledge compared to those trained in other countries. This might be explained by better education in the field of oral health during their specialty training.

Most paediatricians in this study indicated that they would be interested in attending courses/lectures regarding children's oral health. Al-Shunairber et al. reported similar results [17]. An additional research is required to establish an appropriate method to deliver oral health training to practicing paediatricians. For instance, oral health training could be offered to medical students and residents as part of their residency training and continuing medical education, so that they are able to most effectively promote oral health to their patients and refer them to dental care when necessary. There is substantial evidence demonstrating that oral health educational programmes aimed at improving paediatricians' knowledge and practice can be successful [32].

Some of the limitations for the study were identified. Even though respondents' number was enough, they represented only 10% of paediatricians in the UAE. It is likely that those most interested in oral health are overrepresented in the findings of this survey as they were keener on answering the electronic survey. Additionally, the response rate was low. As with any self-administered survey, the answers given may not reflect actual practices and attitudes. In this study, it would have been helpful to ask about the source of oral health education, if any received. It would have also been helpful if we included a question about how paediatricians perceived their current knowledge in order to compare it with their actual knowledge scores.

## 5. Conclusions

Based on this study's results, the following conclusions could be made:

- UAE paediatricians who had more than or equal to 21 years of experience had significantly a better score of knowledge than those with less than 20 years of experience.
- Adequate knowledge was found among UAE paediatricians regarding the need to prescribe antibiotics in case of extraoral cellulitis and intraoral swelling associated with fever. Inversely, insufficient knowledge was demonstrated regarding the treatment of localised dental abscess in the absence of fever.
- Assessment of the association between behaviour, attitude, and score of knowledge in this study revealed that surveyed paediatricians who had better knowledge regarding oral health demonstrated better behaviour scores.

We recommend developing educational programmes for practicing paediatricians in the UAE in the form of continuous educational courses. Incorporation of oral health knowledge in the curricula of the paediatric residency training programmes might be a beneficial method to provide the required information and awareness about oral health for the training paediatricians in the UAE.

## Figures and Tables

**Table 1:** Demographic characteristics, educational level, and occupation of paediatricians.

Gender	No. (%)

Males	102 (55.1)
Females	83 (44.9)
Professional title	
Consultant	57 (30.8)
Specialist	103 (55.7)
Resident	25 (13.5)
Years of experience	
2–5	37 (20)
6–10	46 (24.9)
11–15	41 (22.2)
16–20	30 (16.1)
≥21	31 (16.8)
Practice setting	
Government practice	126 (68.1)
Private practice	47 (25.4)
Others	12 (6.5)
Emirate	
Abu Dhabi	57 (30.8)
Dubai	77 (41.6)
Sharjah	20 (10.9)
Ajman	9 (4.9)
Ras Al-Khaimah	11 (5.9)
Fujairah	11 (5.9)
Um Al Quwain	0
Country of specialty training	
UAE	51 (28)
USA	8 (4.4)
UK	28 (15.4)
Sudan	11 (6.0)
India	21 (11.5)
Jordan	11 (6.0)
KSA	4 (2.2)
Iraq	4 (2.2)
Ireland	3 (1.6)
Germany	6 (3.3)
Egypt	22 (12.1)
Canada	2 (1.1)
Pakistan	5 (2.7)
South Africa	2 (1.1)
Kuwait	1 (0.5)
Syria	2 (1.1)
Yemen	1 (0.5)

**Table 2:** Oral health knowledge questions.

Items		No. (%)

(1) What is the appropriate age for a child's first dental visit?		
6–12 months		**95 (51.4)**
2 years		68 (36.8)
3-4 years		9 (4.9)
There is no specific age		13 (7.0)
(2) Does a child with no cavities need to visit a dentist?		
Yes		**173 (93.5)**
No		12 (6.5)
(3) What is the most appropriate age for children to start brushing their teeth with fluoridated toothpaste?		
As soon as the first tooth erupts (6–12 m)		**77 (41.6)**
After the eruption of primary molars (2-3 yrs)		88 (47.6)
At age 6		8 (4.3)
There is no specific age		12 (6.5)
(4) The best time to give a child a sugary snack is		
In between meals		134 (71.4)
Immediately after a meal		**42 (22.7)**
First thing in the morning		9 (4.9)
(5) Can early carious lesions without cavitation be remineralised or healed?		
Yes		97 (52.4)
No		88 (47.6)
(6) A 5-year-old child attended your clinic with fever, and an extra- and intraoral swelling. The most appropriate treatment is		
Prescribe a course of antibiotics and inform the parents that there is no need for treatment		3 (1.6)
Prescribe a course of antibiotics and refer the child to the dentist immediately		**159 (85.9)**
Prescribe analgesics only		23 (12.4)
(7) A 5-year-old child attended your clinic with a localised dental abscess, and there is no fever. The most appropriate treatment is		
Prescribe a course of antibiotics and inform the parents that there is no need for treatment		0
Prescribe a course of antibiotics and refer the child to the dentist immediately		123 (66.5)
Prescribe analgesics and refer the child to the dentist immediately		**62 (33.5)**
(8) A 10-day-old infant attended your clinic. Upon examination, you noticed the following, the most appropriate diagnosis is		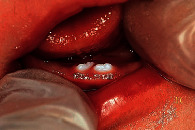
Bohn's nodules		16 (8.6)
Neonatal/natal teeth		**105 (56.8)**
Epstein pearls		9 (4.9)
Normal teething		55 (29.7)
(9) A 9-month-old infant attended your clinic. Upon examination, you noticed the following, the most appropriate diagnosis is		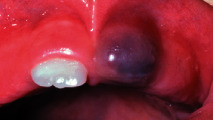
Bohn's nodules		18 (9.7)
Haemangioma		62 (33.5)
Melanoma		9 (4.9)
Eruption cyst/hematoma		**96 (51.9)**

All the correct answers to the questions are in **bold**.

**Table 3:** Correlation between the score of knowledge and paediatricians' demographics.

Variables	*N*	Mean score of knowledge (±SD)	*P* value
Gender			
Male	102	5 (±1.67)	
Female	83	5 (±1.65)	0.897
Title			
Consultant	57	5 (±1.58)	
Resident	25	5 (±1.89)	
Specialist	103	5 (±1.66)	0.904
Years of experience			
2–5	37	5 (±1.87)	
6–10	46	5 (±1.69)	
11–15	41	4 (±1.42)	
16–20	30	5 (±1.69)	
≥21	31	6 (±1.38)	**0.040** ^*∗*^
Practice setting			
Government practice	126	5 (±1.70)	
Private practice	47	5 (±1.68)	
Others	12	5 (±0.96)	0.523
Emirate			
Abu Dhabi	57	5 (±1.83)	
Ajman	9	4 (±1.45)	
Dubai	77	5 (±1.44)	
Fujairah	11	3 (±1.47)	
Ras Al-Khaimah	11	5 (±2.04)	
Sharjah	20	4 (±1.31)	0.104
Specialty training area			
Arab	107	5 (±1.68)	
Western	47	6 (±1.58)	
Indian Subcontinent	28	4.5 (±1.62)	**0.031** ^*∗*^

^*∗*^Statistically significant *P* value as determined by the Mann–Whitney *U* test.

**Table 4:** Paediatricians' behaviour questions.

	No. (%)		No. (%)

(1) Do you routinely examine the oral cavity and dentition of your patients?	**Yes 126 (68.9)**		No 57 (31.1)
(2) Do you include information regarding caries-causing cariogenic food in your dietary counselling to your patients?	Never	Sometimes	**Always**
	21 (11.5)	138 (75.4)	**24 (13.1)**
(3) Do you recommend the use of toothpaste without fluoride for children?	Yes 68 (37.2)		**No 115 (62.8)**
(4) How do you reward your patients?	**No rewards**	Sweets	**Others**
	**33 (18)**	88 (48.1)	**62 (33.9)**

Correct answers are in **bold** font.

**Table 5:** Paediatricians' attitude questions.

	Yes	No
	No. (%)	No. (%)

(1) Do you believe you are able to answer parents' questions about oral health?	**100 (54.6)**	83 (45.4)
(2) Do you believe that toothpaste with fluoride is safe for children below 6?	**93 (50.8)**	90 (49.2)
(3) Do you think that at will or night feeding may affect the teeth?	**163 (89.1)**	20 (10.9)

Correct answers are in **bold**.

## Data Availability

The data used to support this study will be made available from the corresponding author upon request.
